# Lack of association of the *IL‐1RN* and *IL‐10* polymorphisms with risk of psoriasis: A meta‐analysis

**DOI:** 10.1002/mgg3.512

**Published:** 2018-12-06

**Authors:** Ju Qiao, Qian‐Nan Jia, Hong‐Zhong Jin

**Affiliations:** ^1^ Department of Dermatology, Peking Union Medical College Hospital Chinese Academy of Medical Sciences and Peking Union Medical College Beijing China

**Keywords:** interleukin‐1 receptor antagonist, interleukin‐10, polymorphism, psoriasis

## Abstract

**Background:**

The present study carried out a meta‐analysis to investigate whether the interleukin‐1 receptor antagonist (*IL‐1RN*) VNTR polymorphism and three *IL‐10 *single‐nucleotide polymorphisms (SNPs) rs1800896, rs3021097, and rs1800872 are associated with psoriasis risk.

**Methods:**

Wanfang, China National Knowledge Infrastructure, Medline, and PubMed databases were searched for potential studies published until 2 November 2017. Forest plots were generated.

**Results:**

Thirteen case–control studies were included in the review. The results of meta‐analyses revealed no association of the IL‐1RN*2 allele with psoriasis in the overall populations (odds ratio [OR] = 1.16, 95% confidence intervals [CI]: 0.89–1.50, *p* = 0.279), Asians (OR = 1.27, 95% CI: 0.73–2.23, *p* = 0.403), and Caucasians (OR = 1.04, 95% CI: 0.88–1.23, *p* = 0.669). Under the allelic model, there was no statistically significant association of psoriasis with the *IL‐10* SNPs rs1800896 (G allele vs. A allele: OR = 1.03, 95% CI: 0.90–1.18, *p* = 0.639), rs3021097 (C allele vs. T allele: OR = 1.17, 95% CI: 0.88–1.56, *p* = 0.288), and rs1800872 (C allele vs. A allele: OR = 1.01, 95% CI: 0.81–1.25, *p* = 0.951). No publication bias was found by Egger's test and Begg's funnel plots.

**Conclusion:**

Current published studies fail to support an association of the *IL‐1RN *VNTR polymorphism and *IL‐10* SNPs rs1800896, rs3021097, and rs1800872 with psoriasis risk.

## 1 INTRODUCTION

Psoriasis is a common inflammatory disease of the skin affecting 2% of the population (Boehncke & Schon, 2015). Plaque psoriasis, the most common variant of psoriasis, is characterized by inflamed, red skin covered by a silvery white scale (Boehncke & Schon, 2015). Psoriasis is an independent risk factor for mortality and contributes to numerous comorbid conditions, including rheumatological arthritis, depression, cardiovascular disease, and diabetes (Kim, Jerome, & Yeung, 2017). At present, there is no cure for psoriasis and there are no specific markers that can accurately predict the development of psoriasis. Although psoriasis pathogenesis remains poorly understood, it is accepted by most dermatologists that psoriasis arises via the interactions of genetic, immunological, and environmental factors (Mahil, Capon, & Barker, 2016). Currently, more than 40 independent loci are associated with the susceptibility to psoriasis.

Interleukin‐1 receptor antagonist (IL‐1Ra) is a member of the IL‐1 family and an important anti‐inflammatory cytokine. IL‐1Ra competitively blocks the effects of IL‐1αand IL‐1β by binding to the IL‐1 receptor. An inbalance between IL‐1 and IL‐1Ra is associated with increased production of pro‐inflammatory cytokines and the development of inflammatory disorders (Mistry, Savic, & Hilst, 2017). IL‐1Ra‐deficient mice spontaneously developed a dermatitis that histologically resembled human psoriasis (Nakajima et al., 2010; Shepherd, Little, & Nicklin, 2004). In a human coculture model with keratinocytes and autologous T cells, application of a recombinant human form of IL‐1Ra significantly reduced pro‐inflammatory cytokine production (Renne, Schafer, Werfel, & Wittmann, 2010). In addition, the levels of pro‐inflammatory cytokines were significantly up‐regulated in lesional psoriatic epidermis due to decreased formation and secretion of IL‐1Ra (Debets et al., 1997; Kristensen et al., 1992). In intron 2 of the IL‐1Ra gene (*IL‐1RN*), there is an 86‐basepair variable number tandem repeat (VNTR) polymorphism. The *IL‐1RN* allele 2 is thought to result in higher IL‐1Ra release (Bid, Manchanda, & Mittal, 2006). Since IL‐1Ra is involved in the inflammatory responses within psoriatic plaques, dermatologists have carried out genetic studies to assess the relationship of the *IL‐1RN* VNTR polymorphism with psoriasis risk. A quantitative summary of their findings is needed.

IL‐10 is an anti‐inflammatory cytokine synthesized by monocytes, macrophages, and lymphocytes as a response to inflammation (Fontoura et al., 2015). It is able to inhibit antigen presentation and suppress the synthesis and function of a number of pro‐inflammatory cytokines, including tumor necrosis factor‐α (TNF‐α), IL‐6, and IL‐1 (Rutz & Ouyang, 2016). IL‐10 mRNA expression was dramatically decreased in the psoriatic lesions compared with the normal skin tissues (Cheng et al., 2001). In addition, levels of IL‐10‐producing regulatory B cells (B10 cells) were decreased in patients with psoriasis (Hayashi et al., 2017). Given that IL‐10 played a protective role in inflammation development, adoptive transfer of B10 cells effectively suppressed imiquimod‐induced skin inflammation in a mouse model of psoriasis (Yanaba et al., 2013). Moreover, administration of recombinant IL‐10 significantly reduced the severity of psoriasis in humans (Asadullah et al., 1999). Three single‐nucleotide polymorphisms (SNPs) at ‐592A/C (rs1800872), ‐819C/T (rs3021097), and ‐1082A/G (rs1800896) in the *IL‐10* promoter region have recently been evaluated in a number of genetic association studies regarding their relationship with psoriasis susceptibility, but the findings of these studies are contradictory.

Both IL‐1Ra and IL‐10 are important anti‐inflammatory cytokines in psoriasis pathogenesis. Our study seeks to systematically review the literature and meta‐analyze the results of case–control studies on the relation of *IL‐1RN* and *IL‐10 *polymorphisms with psoriasis risk.

## 2 METHODS

### 2.1 Ethical compliance

Ethics approval was waived because this study does not involve any human participants or animals.

### 2.2 Systematic literature search

On 2nd of November 2017, two reviewers carried out independent electronic searches on the Wanfang, China National Knowledge Infrastructure (CNKI), Medline, and PubMed databases covering the entire period of each database. The following algorithm was applied: “(interleukin‐1 OR interleukin‐10 OR cytokine) AND (psoriasis OR genetics OR polymorphism).” Results were limited to studies in English or Chinese, featuring human participants and with abstracts available. We supplemented these searches by searching review articles and reference lists of the included studies. We did not contact study authors to request additional data. Disagreements were solved by discussion and consensus.

### 2.3 Inclusion/exclusion criteria

Studies were assessed against the following inclusion criteria: (a) assessing the relation of the *IL‐1RN* polymorphism or the *IL‐10* polymorphisms (rs1800896, rs3021097, and rs1800872) with psoriasis risk; (b) used validated measures to determine the presence of psoriasis; (c) the study was case–control designed; (d) all data were original; (e) sufficient information on odds ratios (ORs) with their 95% confidence intervals (95% CIs). Exclusion criteria were as follows: (a) not case–control design; (b) not an original paper; (c) performed on animals or human cell lines; (d) studies based on only cases; (e) not offering essential data. Additionally, studies with potentially overlapping cohorts were excluded from the quantitative synthesis. In such cases, we included the study with the largest sample size.

### 2.4 Data extraction

Data extraction was conducted by two investigators (Ju Qiao and Qian‐Nan Jia). The senior author (Hong‐Zhong Jin) was involved in consulting for the eligibility of a study if a divergence between the two data‐extracting investigators existed. From the finally selected papers, data relating to study characteristics for the following variables were extracted: first author, journal name, publication year, location of study, ethnicity, male percentage, number of study subjects, the frequencies of genotypes or alleles in case and control groups, genotyping method, and Hardy–Weinberg equilibrium (HWE) status.

### 2.5 Statistical analyses

All statistical analyses were conducted using Stata version 10.0. To assess the relation of the *IL‐1RN* VNTR polymorphism and the *IL‐10* variants with susceptibility to psoriasis, pooled ORs and their corresponding 95% CIs were assessed. Between‐study heterogeneity was evaluated using Cochrane’s Q statistic. There are two statistical methods for meta‐analysis: the fixed‐effects model and the random‐effects model. If the *Q* test was statistically significant (*p* < 0.10), estimates were pooled using random‐effects models (SanGiovanni, Berkey, Dwyer, & Colditz, 2000). When between‐study heterogeneity was not significant, the overall effects were calculated using the fixed‐effects model (Mantel & Haenszel, 1959). HWE in controls was calculated by chi‐square test. Funnel plots were drawn as a check for potential publication bias. We also used Egger's asymmetry test to evaluate publication bias. The significance level was set at *p* < 0.05, except for test of heterogeneity.

## 3 RESULTS

### 3.1 Study characteristics

**Figure 1:**
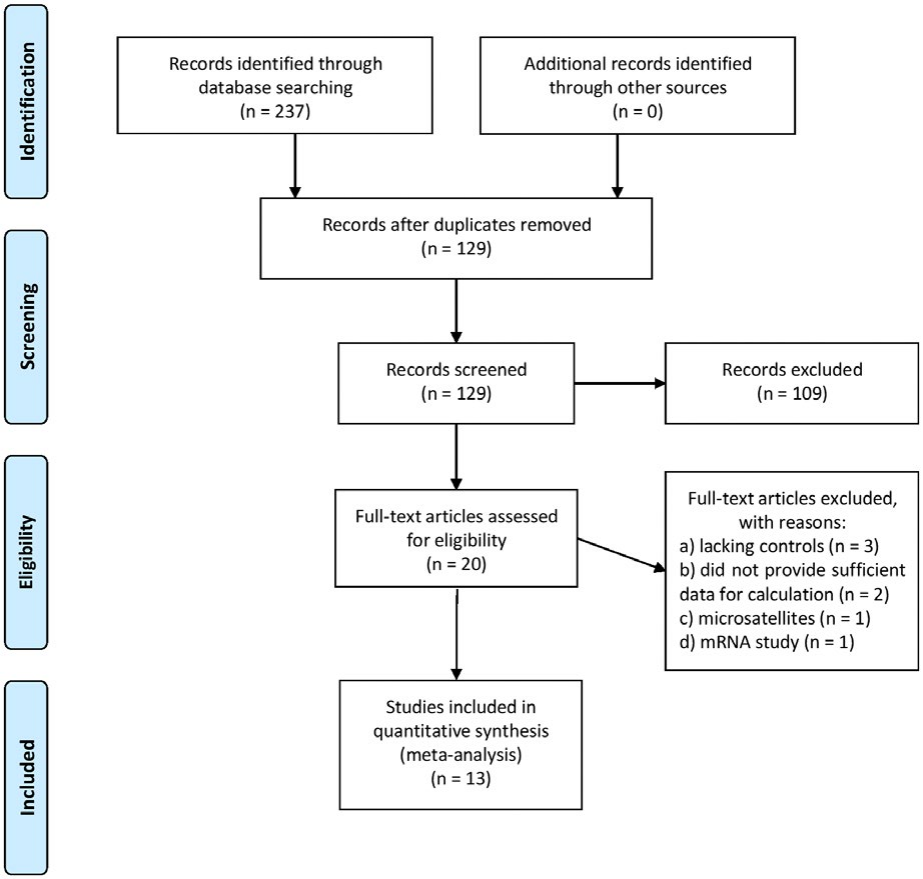
Process of study selection

**Figure 2:**
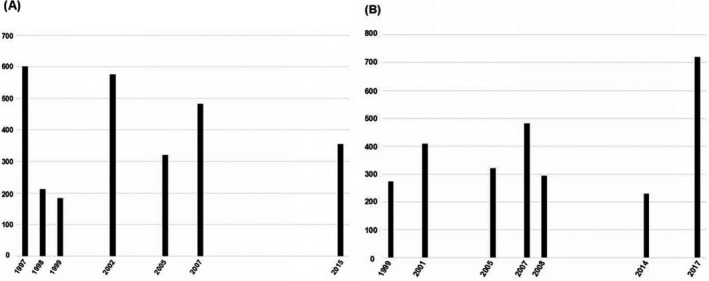
(a) Sample sizes of the studies assessing the interleukin‐1 receptor antagonist variable number tandem repeat polymorphism and risk of psoriasis from 1997 to 2015; (b) Sample sizes of the studies assessing the interleukin‐10 polymorphism rs1800896 from 1999 to 2017

The execution of the search strategy initially resulted in 129 studies after duplicates were removed. Subsequently, 109 studies were excluded at the title/abstract level, and 20 full‐text papers were checked for eligibility. After careful evaluation, a total of 13 studies were included in the review (Chang et al., 2007; Craven et al., 2001; Indhumathi et al., 2017; Karam, Zidan, & Khater, 2014; Li, Dong, Zhu, Yu, & Wang, 1999; Liu, Zhang, Yang, & Xu, 1998; Moorchung, Vasudevan, Chatterjee, Mani, & Grewal, 2015; Peddle, Butt, Snelgrove, & Rahman, 2005; Peng & Wang, 1999; Reich et al., 2002, 1999 ; Tarlow et al., 1997; Wongpiyabovorn et al., 2008). We did not find any additional studies by hand‐searching the reference lists of the included studies. Figure 1 summarizes the process of study selection. The qualified publications included in our meta‐analysis were published between 1997 and 2017. The sample sizes ranged from 60 to 720 participants. Tables 1 and 2 present the characteristics of the individual studies. Figure 2 shows sample sizes of reports over time. We did not find any studies violating HWE in controls.

**Table 1:** Characteristics of the studies evaluating the relationship between the *IL‐1RN* VNTR polymorphism and psoriasis risk

Author	Journal	Year	Country	Ethnicity	Controls	Cases	Male in controls (%)	Male in cases (%)	Genotyping method	Controls selection	HWE
Tarlow	Br J Dermatol	1997	UK and Germany	Caucasians	331	271	Not available	Not available	PCR assay	Not available	Yes
Liu	Natl Med J China	1998	China	Asians	130	82	Not available	56.1	PCR assay	Hospital	Yes
Peng	Immunol J	1999	China	Asians	85	38	Not available	63.2	PCR assay	Population	Yes
Li	J Clin Dermatol	1999	China	Asians	30	30	Not available	53.3	PCR assay	Not available	Yes
Reich	J Invest Dermatol	2002	Germany	Caucasians	345	231	52.8	65.4	PCR assay	Hospital	Yes
Peddle	Ann Rheum Dis	2005	Canada	Caucasians	95	226	Not available	52.2	PCR assay	Not available	Yes
Chang	Br J Dermatol	2007	China	Asians	210	272	Not available	64.7	PCR assay	Hospital	Yes
Moorchung	Indian J Dermatol	2015	India	Asians	243	112	Not available	56.3	PCR assay	Hospital	Yes

Notes

HWE: Hardy–Weinberg equilibrium; IL‐1RN VNTR: interleukin‐1 receptor antagonist variable number tandem repeat; PCR: polymerase chain reaction; UK: United Kingdom.

**Table 2:** Characteristics of the included studies assessing the association between the *IL‐10* polymorphisms and psoriasis risk

Author	Journal	Year	Country	Ethnicity	Controls	Cases	Male in controls (%)	Male in cases (%)	Genotyping method	Selection of controls	IL‐10 polymorphism
Reich	J Invest Dermatol	1999	Germany	Caucasian	123	151	54.5	63.6	Sequencing analysis	Population	rs1800896
Craven	Br J Dermatol	2001	UK	Caucasian	330	78	Not available	41.7	PCR assay	Hospital	rs1800896
Peddle	Ann Rheum Dis	2005	Canada	Caucasian	95	226	Not available	52.2	PCR assay	Not available	rs1800896
Chang	Br J Dermatol	2007	China	Asian	210	272	Not available	64.7	Sequencing analysis	Hospital	rs1800896, rs3021097, rs1800872
Wongpiyabovorn	Clin Exp Dermatol	2008	Thailand	Asian	155	139	16.1	56.8	PCR‐RFLP	Population	rs1800896 and rs1800872
Karam	Cytokine	2014	Egypt	Caucasian	120	110	35.8	24.5	PCR‐RFLP	Not available	rs1800896
Indhumathi	Hum Immunol	2017	India	Asian	360	360	80.0	81.1	TaqMan 5’ allele discrimination assay	Not available	rs1800896 and rs3021097

Notes

IL‐10: interleukin‐10; PCR: polymerase chain reaction; PCR‐RFLP: polymerase chain reaction–restriction fragment length polymorphism; UK: United Kingdom.

### 3.2 No association of IL‐1RN VNTR polymorphism with psoriasis

**Figure 3:**
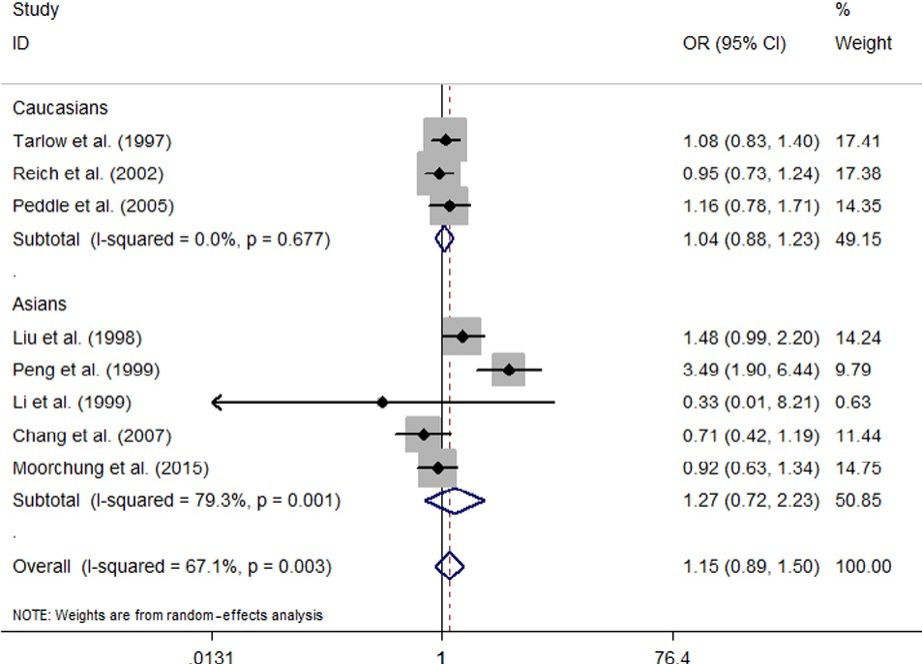
Forest plot of meta‐analysis with data on the association between the interleukin‐1 receptor antagonist variable number tandem repeat polymorphism and risk of psoriasis under allelic model

Five studies evaluating the *IL‐1RN VNTR *polymorphism was performed in Asians (Chang et al., 2007; Li et al., 1999; Liu et al., 1998; Moorchung et al., 2015; Peng & Wang, 1999), and three in Caucasians (Peddle et al., 2005; Reich et al., 2002; Tarlow et al., 1997). Five studies provided genotype data for the polymorphism (Li et al., 1999; Liu et al., 1998; Moorchung et al., 2015; Peng & Wang, 1999; Reich et al., 2002), whereas three studies only contained information on allele frequency (Chang et al., 2007; Peddle et al., 2005; Tarlow et al., 1997). There was significant between‐study heterogeneity (Table 3). Random‐effect meta‐analyses combining the data did not identify any associations of the *IL‐1RN VNTR *polymorphism with psoriasis under four genetic models (dominant model: OR = 1.33, 95% CI: 0.76–1.35, *p* = 0.317; recessive model: OR = 1.77, 95% CI: 0.65–4.82, *p* = 0.262; additive model: OR = 1.80, 95% CI: 0.64–5.09, *p* = 0.266; allelic model: OR = 1.16, 95% CI: 0.89–1.50, *p* = 0.279; Table 3 and Figure 3). Then, subgroups analyses by different ethnicity (Asians and Caucasians) were carried out. The results suggested no significant association between the *IL‐1RN VNTR *polymorphism and psoriasis risk under any of the four genetic models in each ethnic group (Table 3 and Figure 3).

**Table 3:** Summary of results for meta‐analyses

Polymorphism	Dominant model^a^			Recessive model^b^			Additive model^c^			Allelic model^d^		
	OR (95% CI)	*p* value of the z test	*p* derived from heterogeneity test	OR (95% CI)	*p* value of the z test	*p* derived from heterogeneity test	OR (95% CI)	*p* value of the z test	*p* derived from heterogeneity test	OR (95% CI)	*p* value of the z test	*p* derived from heterogeneity test
*IL* *‐1RN* VNTR												
All (*n* = 8)	1.33 (0.76–2.35)	0.317	0.021	1.77 (0.65–4.82)	0.262	0.001	1.80 (0.64–5.09)	0.266	0.005	1.16 (0.89–1.50)	0.279	0.003
Asians (*n* = 5)	1.53 (0.59–3.98)	0.379	0.019	2.38 (0.63–9.02)	0.201	0.003	2.65 (0.55–12.87)	0.226	0.006	1.27 (0.73–2.23)	0.403	0.001
Caucasians (*n* = 3)	0.98 (0.70–1.36)	0.881	Not applicable	0.83 (0.45–1.55)	0.566	Not applicable	0.84 (0.44–1.58)	0.583	Not applicable	1.04 (0.88–1.23)	0.669	0.677
*IL* *‐10* rs1800896												
All (*n* = 7)	1.05 (0.85–1.29)	0.672	0.184	1.18 (0.73–1.89)	0.438	0.098	1.24 (0.76–2.04)	0.395	0.147	1.03 (0.90–1.18)	0.639	0.122
Asians (*n* = 3)	0.96 (0.73–1.27)	0.771	0.278	1.13 (0.56–2.28)	0.733	0.494	1.09 (0.54–2.21)	0.815	0.467	0.93 (0.75–1.16)	0.513	0.276
Caucasians (*n* = 4)	1.17 (0.85–1.61)	0.331	0.123	1.19 (0.60–2.34)	0.623	0.025	1.30 (0.62–2.71)	0.492	0.046	1.10 (0.93–1.31)	0.262	0.111
*IL* *‐10* rs3021097												
All (*n* = 2)	Not applicable	Not applicable	Not applicable	Not applicable	Not applicable	Not applicable	Not applicable	Not applicable	Not applicable	1.17 (0.88–1.56)	0.288	0.092
*IL* *‐10* rs1800872												
All (*n* = 2)	Not applicable	Not applicable	Not applicable	Not applicable	Not applicable	Not applicable	Not applicable	Not applicable	Not applicable	1.01 (0.81–1.25)	0.951	0.895

Notes

CI, confidence interval; IL‐1RN VNTR, interleukin‐1 receptor antagonist variable number tandem repeat; IL‐10, interleukin‐10; OR, odds ratio.

a Dominant model: 22 + 2L versus LL for the *IL‐1RN* VNTR polymorphism; GG + GA versus AA for rs1800896.

b Recessive model: 22 versus 2L + LL for the *IL‐1RN* VNTR polymorphism; GG versus GA + AA for rs1800896.

c Additive model: 22 versus 2L versus LL for the *IL‐1RN* VNTR polymorphism; GG versus GA versus AA for rs1800896.

d Allelic model: 2 allele versus L allele for the *IL‐1RN* VNTR polymorphism; G allele versus A allele for rs1800896; C allele versus T allele for rs3021097; C allele versus A allele for rs1800872.

### 3.3 No association of IL‐10 polymorphisms with psoriasis

**Figure 4:**
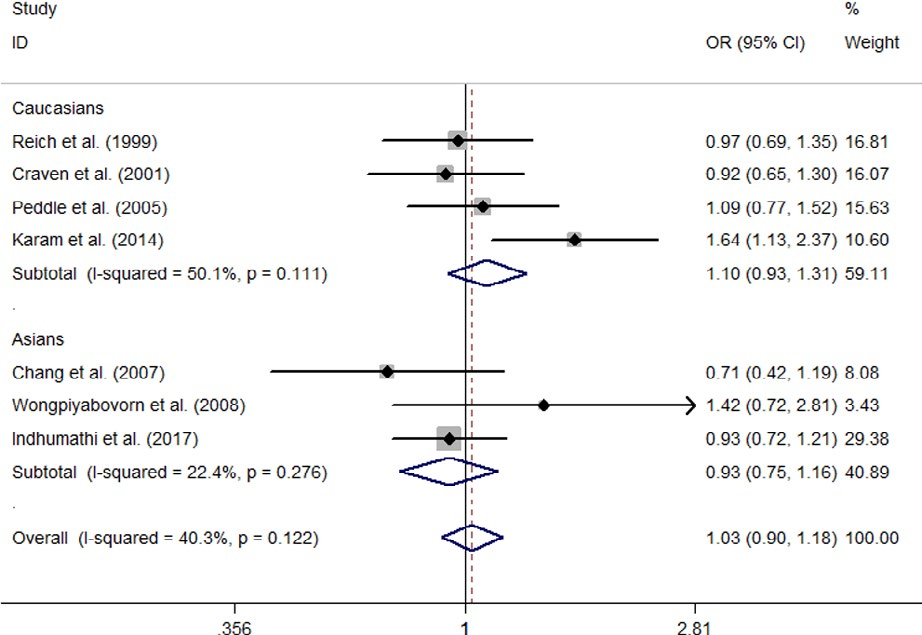
Forest plot of meta‐analysis with data on the association between the interleukin‐10 polymorphism rs1800896 under allelic model

Seven studies provided summaries of data on the *IL‐10* SNP rs1800896 (Chang et al., 2007; Craven et al., 2001; Indhumathi et al., 2017; Karam et al., 2014; Peddle et al., 2005; Reich et al., 1999; Wongpiyabovorn et al., 2008). No evidence for heterogeneity was observed across all studies (Table 3). Pooled results revealed no significant association of rs1800896 with psoriasis risk under any of the comparison models (dominant model: OR = 1.05, 95% CI: 0.85–1.29, *p* = 0.672; recessive model: OR = 1.18, 95% CI: 0.73–1.89, *p* = 0.438; additive model: OR = 1.24, 95% CI: 0.76–2.04, *p* = 0.395; allelic model: OR = 1.03, 95% CI: 0.90–1.18, *p* = 0.639; Table 3 and Figure 4). When studies were subgrouped by ethnicity, the relationship between rs1800896 and psoriasis was not significant in Asian or Caucasian populations (Table 3 and Figure 4).

Two studies investigated the relation between the *IL‐10* SNP rs3021097 (Chang et al., 2007; Indhumathi et al., 2017) and psoriasis risk. Under allelic contrast, the summary OR was 1.17 (95% CI: 0.88–1.56, *p* = 0.288), suggesting no significant association with psoriasis (Table 3). Significant heterogeneity was identified (Table 3).

Two studies evaluated the association of the *IL‐10* SNP rs1800872 with psoriasis risk using data on allelotype (Chang et al., 2007; Wongpiyabovorn et al., 2008). Meta‐analysis of fixed‐effects model provided an estimated OR of 1.01 (95% CI: 0.81–1.25, *p* = 0.951) revealing no relationship between this polymorphism and psoriasis (Table 3). We did not identify between‐study heterogeneity (Table 3).

### 3.4 Sensitivity analysis and publication bias

**Figure 5:**
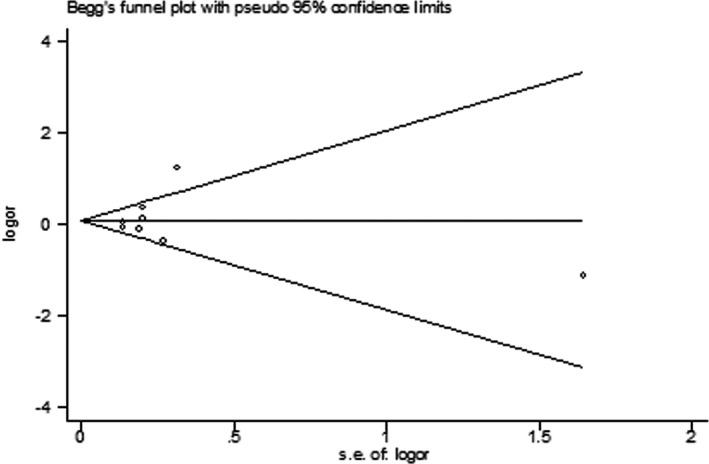
Begg's funnel plot for meta‐analysis of the interleukin‐1 receptor antagonist variable number tandem repeat polymorphism

**Figure 6:**
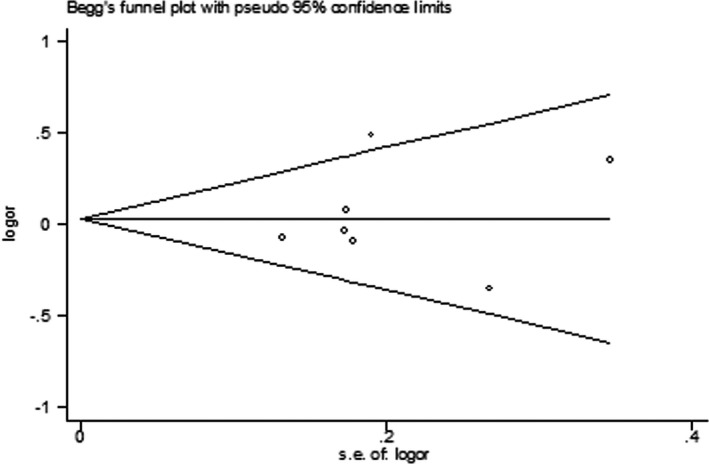
Begg's funnel plot for meta‐analysis of the interleukin‐10 polymorphism rs1800896

We conducted sensitivity analyses to evaluate the stability of the overall effect by removing one study at a time and estimating the summary ORs for the remaining studies. The results remained essentially unchanged for the *IL‐10* polymorphism rs1800896 and the *IL‐1RN* VNTR polymorphism (data not shown). Given that the meta‐analyses for the *IL‐10* SNPs rs3021097 and rs1800872 were performed on a small number of small studies, sensitivity analysis was not carried out for these two SNPs. Begg's funnel plots (Figures 5 and 6) and Egger's test revealed no publication bias (*p* > 0.05 for each polymorphism).

## 4 DISCUSSION

Interleukin‐1Ra and IL‐10 are critical negative regulators of the inflammatory response. Numerous studies have shown the effects of IL‐1Ra on blocking the activity of IL‐1α and IL‐1β in various in vitro and in vivo systems (Paolo & Shayakhmetov, 2016). IL‐1Ra knockout mice develop skin inflammation with histopathological features resembling human psoriasis (Nakajima et al., 2010; Shepherd et al., 2004), while administration of recombinant IL‐1Ra shows beneficial effects in patients with psoriasis (Viguier, Guigue, Pages, Smahi, & Bachelez, 2010). IL‐10 has a broader spectrum of anti‐inflammatory activities because it inhibits the production of a number of pro‐inflammatory cytokines, including interferon‐γ, IL‐8, IL‐6, IL‐1α, and IL‐1β. IL‐10 also up‐regulates the formation and release of anti‐inflammatory molecules. Treatment with recombinant IL‐10 has been shown to be effective in reducing the inflammatory reactions and disease severity in psoriasis patients (Asadullah et al., 1999; Reich et al., 2001). Because IL‐1ra and IL‐10 play an important role in controlling inflammation, it is possible that genetic variants in the *IL‐1RN *and *IL‐10 *genes may contribute to psoriasis susceptibility.

In this meta‐analysis, we systematically reviewed case–control studies on the association between psoriasis risk and genetic variants in the *IL1‐RN* and *IL‐10* genes. The results of our meta‐analyses found no significant effect of the four individual polymorphisms (*IL‐1RN* VNTR polymorphism and *IL‐10* SNPs rs1800896, rs3021097 and rs1800872) on psoriasis risk.

There is no previous meta‐analysis assessing the relation of the *IL‐1RN* VNTR polymorphism with psoriasis risk. Among the eight case–control studies on this polymorphism, conflicting results were reported. Three studies reported an association between this polymorphism and psoriasis (Liu et al., 1998; Peng & Wang, 1999; Tarlow et al., 1997), but the others did not find any associations (Chang et al., 2007; Li et al., 1999; Moorchung et al., 2015; Peddle et al., 2005; Reich et al., 2002). It is unclear what factors contribute to the conflicting results reported in these studies. Differences in genetic background, percentage of men, origin of controls, sample size, and environmental factors may be responsible for the inconsistent results. Meta‐analysis is a quantitative statistical analysis for synthesizing research results across different studies into an overall summary. Our study would benefit clinician by providing a summary of medical literature on the relationship of the *IL‐1RN* VNTR polymorphism with psoriasis. Based on a combined estimate of data from eight genetic studies involving 1,469 psoriasis patients and 1,262 control subjects, we found no association of the *IL‐1RN* VNTR polymorphism with psoriasis risk.

A previous meta‐analysis by Lee, Choi, Ji, and Song, (2012 assessed the *IL‐10* SNPs and psoriasis risk in 2012. In their paper, Lee et al. did not reveal significant associations of rs1800896 and rs3021097 with psoriasis risk in the overall populations, but they identify a significant association between rs1800896 and psoriasis risk in Asian subjects. The SNP rs1800872 was not evaluated in their meta‐analysis. Lee et al. included two case–control studies for evaluating the rs1800896 polymorphism in Asians. In addition to the two studies Lee et al. used, we added a recently published Asian study with a large sample size (720 participants) into the pooled analysis, finding no significant association between rs1800896 and psoriasis in Asians. Given that the meta‐analysis by Lee et al. only included a small number of studies with small sample sizes for evaluating the relation of rs1800896 and psoriasis risk in Asians, the positive association they reported may be unreliable and needs to be confirmed by studies using larger sample size.

Finally, the limitations of our study must be discussed. First, haplotype analysis for the *IL‐10* polymorphisms was not performed because of limited number of studies (*n* = 2) and discrepancy in study methods (Al‐Heresh et al., 2002; Baran, Szepietowski, Mazur, & Baran, 2008). It was noted that the two studies evaluating *IL‐10* haplotype and psoriasis risk did not reported a significant association. Second, owing to low sample size of studies, the association of the *IL‐1RN* VNTR polymorphism and *IL‐10 *SNPs with psoriasis subtypes was not taken into account in our meta‐analysis. Third, there were discrepancies in the presentation of published data for the *IL‐1RN* VNTR polymorphism. It is recommended that further studies should provide data for both genotype and allelotype frequencies of the polymorphism.

In conclusion, current published studies fail to support the hypothesis that the *IL‐1RN* VNTR polymorphism and three common *IL‐10* SNPs rs1800896, rs3021097, and rs1800872 are associated with psoriasis risk.

## CONFLICTS OF INTEREST

The authors declare no conflict of interest.
